# Specialized DNA Structures Act as Genomic Beacons for Integration by Evolutionarily Diverse Retroviruses

**DOI:** 10.3390/v15020465

**Published:** 2023-02-07

**Authors:** Hinissan P. Kohio, Hannah O. Ajoge, Macon D. Coleman, Emmanuel Ndashimye, Richard M. Gibson, Eric J. Arts, Stephen D. Barr

**Affiliations:** Department of Microbiology and Immunology, Schulich School of Medicine and Dentistry, Western University, Dental Sciences Building Room 3007, London, ON N6A 3K7, Canada

**Keywords:** retroviruses, HIV, integration, non-B DNA, slipped DNA, integration hotspots, genome

## Abstract

Retroviral integration site targeting is not random and plays a critical role in expression and long-term survival of the integrated provirus. To better understand the genomic environment surrounding retroviral integration sites, we performed a meta-analysis of previously published integration site data from evolutionarily diverse retroviruses, including new experimental data from HIV-1 subtypes A, B, C and D. We show here that evolutionarily divergent retroviruses exhibit distinct integration site profiles with strong preferences for integration near non-canonical B-form DNA (non-B DNA). We also show that in vivo-derived HIV-1 integration sites are significantly more enriched in transcriptionally silent regions and transcription-silencing non-B DNA features of the genome compared to in vitro-derived HIV-1 integration sites. Integration sites from individuals infected with HIV-1 subtype A, B, C or D viruses exhibited different preferences for common genomic and non-B DNA features. In addition, we identified several integration site hotspots shared between different HIV-1 subtypes, all of which were located in the non-B DNA feature slipped DNA. Together, these data show that although evolutionarily divergent retroviruses exhibit distinct integration site profiles, they all target non-B DNA for integration. These findings provide new insight into how retroviruses integrate into genomes for long-term survival.

## 1. Introduction

The family *Retroviridae* is divided into two subfamilies called *Orthoretrovirinae* and *Spumaretrovirinae*. *Alpha*-, *Beta*-, *Gamma*-, *Delta*-, *Epsilon-retrovirus* and *Lentivirus* represent the six genera of the *Orthoretrovirinae*, and *Bovispumavirus*, *Equispumavirus*, *Felispumavirus*, *Prosimiispumavirus* and *Simiispumavirus* represent the five genera of *Spumaretrovirinae*. Following entry of a retrovirus into cells, the viral RNA genome is converted into double-stranded DNA that associates with several viral and host proteins to form a pre-integration complex (PIC). Soon after completion of DNA synthesis, the viral DNA ends are primed by the enzyme integrase in a process called 3′ end processing. Following docking with the host’s genomic DNA, the viral DNA undergoes a strand transfer reaction resulting in the insertion of the retroviral genome into the host genomic DNA. Host repair enzymes are then thought to complete integration [1]. This insertion of the retroviral genome into the host genome results in a persistent life-long infection.

Selection of integration sites in the host genome by the PIC is not random. The Gamma- and Delta-retroviruses (e.g., murine leukemia virus (MLV), human T cell leukemia virus type 1 (HTLV-1)) and foamy virus (FV) favor integration around transcription start sites (TSS) [2,3,4]. Alpharetroviruses (e.g., avian sarcoma leukosis virus (ASLV)) show no strong preferences, with integration only slightly favored in transcription units or the 5′ end of genes [5,6,7,8]. Lentiviruses, such as human immunodeficiency virus type 1 (HIV-1), simian immunodeficiency virus (isolated from a pig-tailed macaque) (SIV) and feline immunodeficiency virus (FIV) strongly favor integration in active transcription units [9,10,11]. HIV-1 integration sites are the most characterized sites of all retroviruses and have been shown to be associated with regions of high G/C content, high gene density, short introns, high frequencies in short interspersed nuclear elements (SINEs) (e.g., Alu repeats), low frequencies in long interspersed nuclear elements (LINEs) and characteristic epigenetic modifications [9,12].

Thus far, integration site analyses have mostly been conducted on HIV-1 subtype B infections. Based on phylogenetic analyses of full-length genomic sequences, HIV-1 isolates are classified into four distinct groups: group M, N, O and P [13]. HIV-1 M group accounts for the majority of the global pandemic and is subdivided into ten subtypes (A, B, C, D, F, G, H, J, K and L). Additionally, several circulating recombinant forms (CRFs) and unique recombinant forms (URFs) have been identified, which are the result of a recombination event between two or more different subtypes. HIV-1 geographical prevalence is extremely diverse. Subtype C represents approximately 47% of infections worldwide and is prevalent in Africa and Asia. Subtype B infections are common in the Americas, Europe, Australia and part of South Asia, Northern Africa and the Middle East. Subtypes A, D, F, G, H, J, K and L occur mostly in Sub-Saharan Africa, whereas infections with groups N, O and P have been found in confined regions of West-Central Africa.

Several models, not mutually exclusive, have been proposed to explain integration site selection. In the chromatin accessibility model, the structure of chromatin influences accessibility of target DNA sequences to PICs. Target DNA in vivo is not expected to be naked but rather wrapped in nucleosomes. Wrapping target DNA in nucleosomes does not reduce integration, but instead creates hotspots for integration at sites of probable DNA distortion [14,15]. Distortion of DNA in several other protein-DNA complexes has also been shown to favor integration in the major grooves facing outwards from the nucleosome core [16,17]. Although chromatin structure can facilitate integration, chromatin accessibility cannot solely explain the differences observed in integration site preferences.

The protein tethering model suggests that a cellular protein, potentially specific for each retroviral genera, acts as a tethering factor between chromatin and the PIC. The most characterized tethering factor identified to date is lens epithelium-derived growth factor and co-factor p75 (LEDGF/p75) (also known as PSIP1/p75) [18,19,20]. LEDGF/p75 interacts with HIV integrase and tethers the PIC to genomic DNA in transcriptionally active genes marked by specific histone modifications, such as H3K20me1, H3K27me1 and H3K36me3. In similar fashion, the host protein bromodomain/extraterminal domain proteins (BETs) bound to acetylated histones (e.g., H327ac and H3K9ac) interact with MLV integrase and tether the PIC to genomic DNA in transcriptionally active promoters, enhancers and super enhancers [21,22,23,24]. LEDGF/p75 and BET depletion studies demonstrated that integration still occurs, but with reduced efficiency and an altered integration site selection profile [20,22,23,24,25,26,27,28]. Several cellular proteins have been proposed to facilitate integration, including barrier to autointegration factor (BAF), high mobility group A1 (HMGA1), integrase interactor 1 (Ini-1), and heat shock protein 60 (Hsp60) [29,30,31]. Some of these proteins might contribute to the PIC function by coating and condensing the viral DNA, thereby assisting the assembly of the viral nucleoprotein complexes. Several cellular chromatin proteins have also been suggested to influence integration, such as Ini-1, EED, SUV39H1 and HP1γ (reviewed in references [12,32,33]). Cleavage and polyadenylation specificity factor 6 (CPSF6) facilitates HIV-1 PIC import and helps direct the PIC to the nuclear interior to locate gene-dense euchromatin for integration [34,35,36,37,38]. Recently, we identified apolipoprotein B editing complex (APOBEC3) as a potential new host factor that also influences integration site selection by promoting a more transcriptionally silent integration site profile [39]. Moreover, just as DNA-binding proteins can promote integration, they can also block access of integration complexes, creating regions refractory for integration [16,40,41]. Binding to different factors or a differential binding affinity to a factor by the PIC could modulate integration site selection between lentivirus types or even between different HIV-1 subtypes.

Previous analyses of primary DNA sequences (~20 base pairs (bp)) flanking integration sites only revealed a weak consensus motif [42]. We previously assessed a broader window of a primary sequence (80 bp) around HIV-1 integration sites and discovered that HIV-1 integration sites were highly enriched near specialized genomic features called non-B DNA [43]. Under certain conditions, non-B DNA can form functional secondary structures in the human genome based on specific nucleotide sequences that exhibit non-canonical DNA base pairing. The formation of these secondary structures is dependent on a number of factors, such as local sequence features including symmetry, repetitive tracts and GC content, and other factors, such as interactions with DNA binding proteins, DNA unwinding, superhelical status of the DNA and the presence of additional nucleic acid strands. At least 10 non-B DNA conformations are identified, including inverted repeats, direct repeats, mirror repeats, short-tandem repeats, guanine-quadruplex (G4), A-phased, cruciform, slipped, triplex and Z-DNA [44,45]. Recently, we showed that G4 DNA and other non-B DNA motifs influence productive and latent HIV-1 integration, further highlighting the potential importance of these genomic features for HIV-1 infection [46].

Here, we present a meta-analysis of previously published integration site datasets for HIV-1 subtype B, SIV, FIV, HTLV-1, FV, MLV, ASLV and MMTV, with the inclusion of new experimental data from HIV-1 subtypes A, C and D. We present for the first time the non-B DNA integration site profiles of these evolutionarily diverse retroviruses and identify striking similarities and differences in preferences for non-B DNA between the different retroviruses.

## 2. Materials and Methods

Ugandan and Zimbabwean cohort description. Details pertaining to the Uganda study population have been reported previously [47,48,49,50]. Briefly, women who became HIV infected while participating in the Hormonal Contraception and Risk of HIV Acquisition Study in Uganda were enrolled upon primary infection with HIV-1 into a subsequent study, the Hormonal Contraception and HIV-1 Genital Shedding and Disease Progression among Women with Primary HIV Infection (GS) Study. Ethical approval was obtained from the institutional review boards (IRBs) from the Joint Clinical Research Centre and UNST in Uganda, from the University of Zimbabwe, from the University Hospitals of Cleveland, and recently, from Western University. All adult subjects provided written informed consent and no child participants were included in the study. Blood and cervical samples were collected every month for the first six months, then every three months for the first two years, and then every six months up to 9.5 years. Women who had CD4 lymphocyte counts of 200 cells/mL and/or who developed severe symptoms of HIV infection (WHO clinical stage IV or advanced stage III disease) were offered combination antiretroviral therapy (cART) and trimethoprim-sulfamethoxazole (for prophylaxis against bacterial infections and *Pneumocystis jeroveci* pneumonia).

DNA Isolation. Total genomic DNA was extracted from PBMCs isolated from the infected individuals using the QIAmp DNA mini kit (Qiagen) and processed for integration site analysis in a DNA clean room using different pipets, as previously described in detail [43,51]. Briefly, the DNA was subjected to MseI/SacI digestion and linker ligation. Following purification, the DNA was subjected to two rounds of nested PCR using 3′ LTR- and linker-specific primers. Samples were sequenced using the paired-end Illumina MiSeq platform (San Diego, CA, USA) at the London Regional Genomics Centre/Robarts Research Institute (Western University, London, ON, Canada) and Case Western Reserve University (USA).

HIV-1 integration site library. Integration sites for the HIV-1 subtype B in vitro and in vivo datasets were obtained directly from the Retrovirus Integration Database (RID) v2.0 (Hg19) [https://rid.ncifcrf.gov/index.php] (accessed September 12, 2022). HIV-1 subtype A, C and D integration sites were newly obtained from infected individuals in Uganda and Zimbabwe. Integration sites were determined from the sequence junction between the HIV-1 3′ LTR and human genome sequences. Each paired fastq sequencing read was quality trimmed and excluded from further analysis if the LTR-genome junction sequence did not match between the two paired reads. The HIV-1 LTR-containing fastq sequences were filtered by allowing up to a maximum of five mismatches with the reference NL4-3 LTR sequence. LTR sequences matching any region of the human genome (GRCh37/hg19) were discarded. Flanking human genomic sequences more than 20 nucleotides in length were used to identify integration sites using our in-house bioinformatics pipeline (Barr Lab Integration Site Identification Pipeline (BLISIP version 2.9)) [39,43,46]. BLISIP version 2.9 includes the following updates: BEDtools (v2.25.0), bioawk (awk version 20110810), bowtie2 (version 2.3.4.1), and restrSiteUtils (v1.2.9). All genomic sites within each dataset that hosted two or more sites (i.e., identical sites) were collapsed into one unique site for the analysis. Sites that could not be unambiguously mapped to a single region in the genome were excluded from analysis. All non-B DNA motifs were defined according to previously established criteria [52]. Lamina-associated domains (LADs) were retrieved from http://dx.doi.org/10.1038/nature06947 [53]. For each dataset that used restriction enzymes for the preparation of their libraries, restriction enzyme site-matched random control integration sites were independently generated by matching each experimentally determined site with 50 random sites in silico that were constructed to be the same number of bases from the restriction site as was the experimental site, as previously described [54]. Random control datasets for the libraries generated by random fragmentation (e.g., shearing) were generated using the random tool of BEDTools v2.28 [55]. The integration site heatmaps were generated using our in-house Python program BLISIP Heatmap (BLISIPHA v1.0), which calculates the fold enrichment of sites in each distance bin for each feature, compared to that of the appropriate control dataset.

Statistical Analyses. Differences in the integration site profile data were tested for its statistical significance using Fisher’s exact tests (two-sided). *p* values less than 0.05 were considered significant. Analyses were performed using Graphpad Prism 9 version 9.4.1 (Graphpad Software, San Diego, CA, USA). Pairwise analyses were performed on the retroviral integration site profile preferences (fold enrichment and depletion values) using the Euclidean distance as the measurement method (Heatmapper) [56].

## 3. Results

### 3.1. Integration Site Dataset Acquisition and Analyses

The integration site profiles of evolutionarily diverse retroviruses with respect to non-B DNA was previously unknown. To compare integration site profiles of HIV-1, SIV, FIV, HTLV-1, FV, MLV, ASLV and MMTV, we analyzed 118,020 unique integration sites from previously published datasets (Table S1). To allow for a more comparable analysis of integration site profiles, we updated these integration site profiles, some of which are over 20 years old, using the human genome assembly GRCh37/19. Our update included an assessment of several genomic features, some of which were not included in earlier retroviral integration site studies: CpG islands, DNAseI hypersensitivity sites (DHS), ERVs, heterochromatic DNA regions (e.g., lamina-associated domains (LADs) and satellite DNA), SINEs, LINEs, low complexity repeats (LCRs), oncogenes, genes, simple repeats and TSS. In addition, we measured integration site enrichment near the following non-B DNA features: A-phased motifs, cruciform motifs, direct repeats, G4 motifs, inverted repeats, mirror repeats, short tandem repeats, slipped motifs, triplex motifs and Z-DNA motifs. Our analyses focused on unique integration sites. Sites falling in repeat regions that could not be unambiguously mapped to a single region in the genome and regions that could not be confidently placed on a specific chromosome (e.g., ChrUn) were excluded from analyses. Enrichment of integration sites within genomic features was determined by comparing the proportion of sites with either a restriction enzyme-matched random control (MRC) to account for restriction enzyme site bias in the cloning procedure during library construction, or a random control (RC) for comparison of datasets that used DNA shearing/fragmentation during library construction (see Materials and Methods) (Table S1).

### 3.2. Evolutionarily Divergent Retroviruses Exhibit Distinct Integration Site Profiles

**Figure 1:**
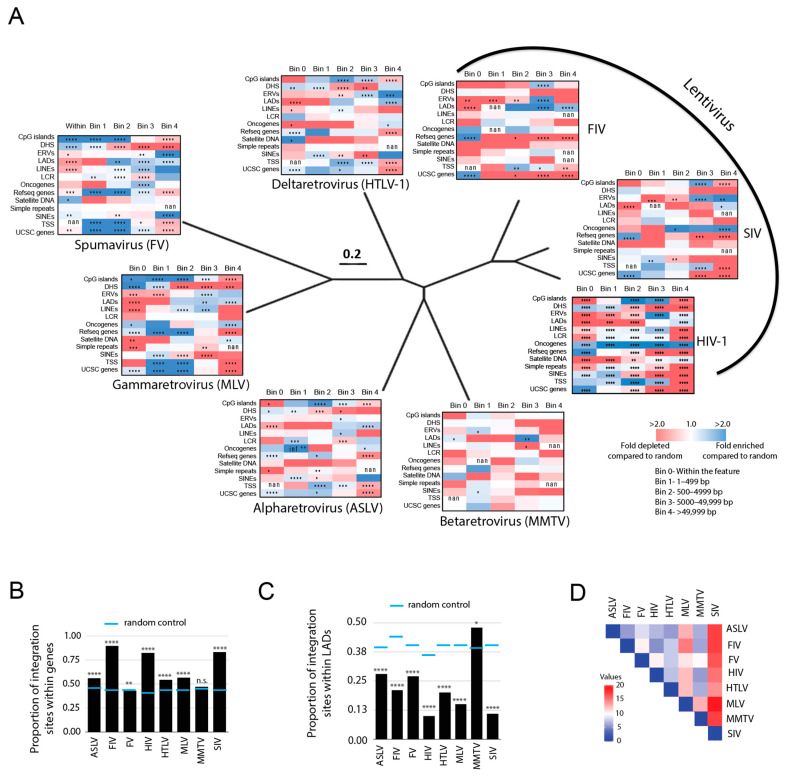
Evolutionarily diverse retroviruses exhibit distinct integration site preferences. (**A**) Heatmaps depicting the fold enrichment or depletion of integration sites near common genomic features compared to matched random controls. Darker shades represent larger fold-changes in the ratio of integration sites to matched random control sites. Blue color indicates enriched sites, red for depleted). Bins represent the distance of the integration sites from each genomic feature. Bin 0 = within the feature; Bin 1 = 1–499 bp; Bin 2 = 500–4999 bp; Bin 3 = 5000–49,999 bp; Bin 4 = >49,999 bp away from the feature. Heatmaps of the diverse retrovirus genera were superimposed on a BioNJ tree constructed using their reverse transcriptase amino acid sequences using the Dayhoff substitution model with 1000 bootstraps. All branches are scaled according to the number of amino acid changes per site. The phylogenetic tree shows the evolutionary relatedness of the different retrovirus genera only. Significant differences are denoted by asterisks (*p < 0.05; ** *p* < 0.01; *** *p* < 0.001; **** *p* < 0.0001) (Fisher’s exact test, two-tailed). HIV-1 = human immunodeficiency virus, SIV = simian immunodeficiency virus (isolated from a pig-tailed macaque), FIV = feline immunodeficiency virus, HTLV-1 = human T-lymphotrophic virus Type 1, MLV = murine leukemia virus, FV = foamy virus, ASLV = avian sarcoma leucosis virus, MMTV = mouse mammary tumor virus. (**B**) Proportion of the retroviral integration sites located within genes, compared to the random control (blue lines). (**C**) Nuclear localization of integration sites was determined by quantifying the proportion of total integrations that fell within a lamin-associated domain (LAD) (=1) as opposed to outside an LAD (=0). (**D**) Pairwise analysis was performed on the retroviral integration site profile preferences (based on fold enrichment and depletion values within 5000 bp of each feature) using the Euclidean distance as the measurement method (Heatmapper) [56]. Weaker relationships between retroviral integration site profiles are indicated by darker red color in the pairwise distance matrix, whereas stronger relationships are indicated by darker blue color. * *p* < 0.05; ** *p* < 0.01; *** *p* < 0.001; **** *p* < 0.0001; n.s., not significant; Fisher’s exact test, two-sided. Infinite number (inf), 1 or more integrations were observed when 0 integrations were expected by chance. Not a number (nan), 0 integrations were observed and 0 were expected by chance.

Integration sites were quantified and placed in five bins based on their distance from each genomic feature (within the feature, 1–499 bp, 500–4999 bp, 5000–49,999 bp and >49,000 bp). Heatmaps from each retrovirus showing the fold-enrichment and fold-depletion in each bin were compared to MRC or RC (Figure 1A). Consistent with previous studies [9,10,11], HIV-1, SIV and FIV integration sites are significantly enriched within genes (83%, 84%, and 90%, respectively) (*p* < 0.0001) (Figure 1A,B, Table S2). Our analysis of HTLV-1, ASLV and MLV also agreed with previous reports, confirming that these viruses exhibit only modest preferences for integration within genes with 54%, 56% and 56% of integration sites found within genes, respectively (Figure 1A,B, Table S2) [57,58]. In contrast, MMTV and FV showed no preference for integration into genes (47% and 43%, respectively, compared to 47% for the random control). No retrovirus showed a preference for integration directly into TSS; however, MLV and FV showed significant enrichment of integration sites within 500 bp of TSS and CpG islands (*p* < 0.0001) (Figure 1A, Table S2). Repetitive elements, such as LINEs, SINEs, ERVs (e.g., retrotransposons), satellite DNA, simple repeats (e.g., microsatellites) and LCRs account for nearly half of the human genome sequence. However, no strong preference for integration into these regions was observed for any of the retroviruses except for HIV-1 and FV which targeted SINEs, and FV, MMTV and HTLV-1 which targeted satellite DNA (Figure 1A). It is known that the nuclear architecture influences HIV-1 integration site selection and proviral expression [53,59]. HIV-1 strongly disfavors integration into heterochromatin positioned in LADs at the nuclear periphery; although some integration does occur in these regions, contributing to the latent reservoir [60]. As with HIV-1, most retroviruses significantly disfavored integration into LADs with only 10–28% of sites falling within LADs (Figure 1C, Table S2). In stark contrast, 48% of MMTV integration sites were significantly enriched in LADs. Pairwise analysis of the different integration site profile preferences overall (based on fold enrichment and depletion values within 5000 bp of each feature) showed that SIV and MLV had the least similarity to the other retroviruses (Figure 1D).

### 3.3. Evolutionarily Divergent Retroviruses Target Non-B DNA for Integration

**Figure 2:**
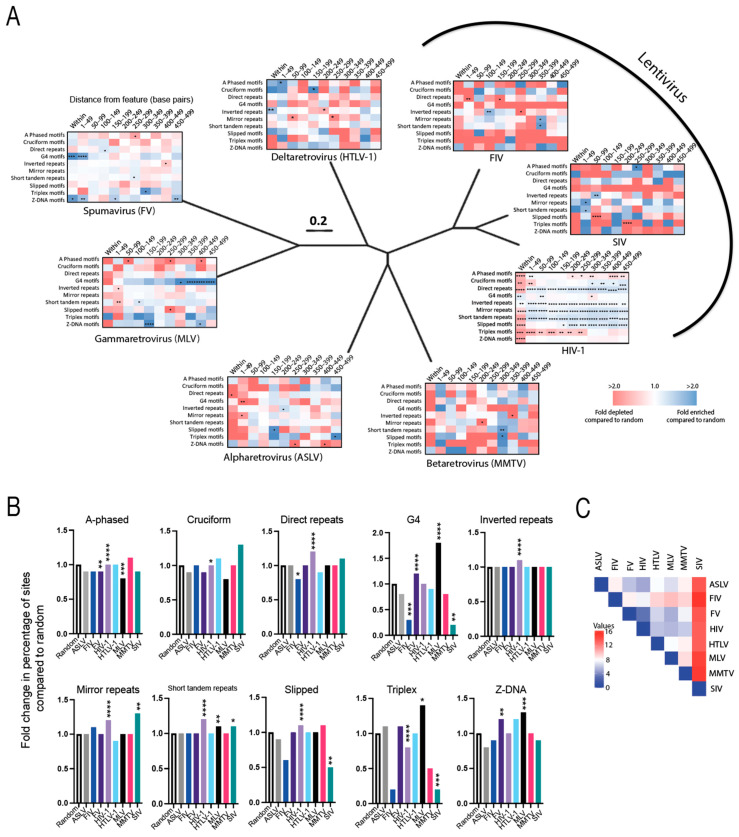
Evolutionarily diverse retroviruses target non-B DNA for integration. (**A**) Heatmaps illustrating the fold-enrichment or -depletion of unique retroviral integration sites near non-B DNA features compared to matched random controls. Darker shades represent larger fold-changes in the ratio of integration sites to matched random control sites. Blue color indicates enriched sites, red for depleted). The distance in base pairs away from the non-B DNA features are shown above each heatmap. Heatmaps of the diverse retrovirus genera were superimposed on a BioNJ tree constructed using their reverse transcriptase amino acid sequences using the Dayhoff substitution model with 1000 bootstraps. All branches are scaled according to number of amino acid changes per site. The phylogenetic tree shows the evolutionary relatedness of the different retrovirus genera only. (**B**) Fold change in the percentage of integration sites within 500 bp of various non-B DNA compared to random. (**C**) Pairwise analysis was performed on the retroviral integration site profile preferences (based on fold enrichment and depletion values within 500 bp of each feature) using the Euclidean distance as the measurement method (Heatmapper) [56]. Weaker relationships between retroviral integration site profiles are indicated by darker red color in the pairwise distance matrix, whereas stronger relationships are indicated by darker blue color. Significant differences are denoted by asterisks * *p* < 0.05; ** *p* < 0.01; *** *p* < 0.001; **** *p* < 0.0001) (Fisher’s exact test, two-sided).

Non-B DNA is a genomic correlate of HIV-1 integration site selection, but its influence on integration site targeting of other retroviruses was previously unknown [39,43,46]. Analysis of the different retroviral integration site profiles showed that all retroviruses exhibited enriched integration within 500 bp of non-B DNA (Figure 2A,B and Table S3). A 500 bp window was selected for its potential functional significance, as previously described [46]. Comparison of the percentage of sites falling within 500 bp of the non-B DNA to that of a random distribution revealed stark differences among the different viruses. Notably, HIV-1 exhibited enrichment near direct repeats, inverted repeats, mirror repeats, short tandem repeats and slipped DNA (*p* < 0.0001); ASLV near triplex DNA (*p* > 0.05); FIV near mirror repeats (*p* > 0.05); FV near G4 motifs and Z-DNA (*p* < 0.01); HTLV-1 near cruciform and Z-DNA (*p* > 0.05); MLV near G4 motifs, short tandem repeats, triplex and Z-DNA (*p* < 0.05); MMTV near A-phased motifs and slipped motifs (*p* > 0.05); and SIV near cruciform motifs (*p* > 0.05), mirror repeats and short tandem repeats (*p* < 0.05) (Figure 2B and Table S3). Of note, integration was disfavored within 500 bp of non-B DNA for several viruses. HIV-1 disfavored triplex motifs (*p* < 0.0001); ASLV disfavored A-phased, cruciform, G4, slipped and Z-DNA motifs (*p* > 0.05); FIV disfavored direct repeats and G4 motifs (*p* < 0.05), and A-phased, slipped, triplex and Z-DNA motifs (*p* > 0.05); FV disfavored A-phased motifs (*p* < 0.01) and cruciform motifs (*p* > 0.05); HTLV-1 disfavored direct repeats, G4 motifs and mirror repeats (*p* > 0.05); MLV disfavored A-phased motifs (*p* < 0.001) and cruciform motifs (*p* > 0.05); MMTV disfavored G4 and triplex motifs (*p* > 0.05); and SIV disfavored A-phased and Z-DNA motifs (*p* > 0.05), and G4, slipped and triplex motifs (*p* < 0.01) (Figure 2B and Table S3).

We have previously observed that the distribution of integration sites within the 500 bp window can vary significantly with strong enrichment observed at discrete distances away from the non-B DNA feature [39,46]. Analysis of the distribution of integration sites in 50 bp intervals within the 500 bp window showed that FV, HTLV-1, SIV and HIV-1 exhibited an enrichment of sites directly in or within 50 bp of multiple non-B DNA features, whereas FIV, MMTV, ASLV and MLV tended to integrate more distal (100–500 bp) to the features (Figure 2A and Table S3). Notably, HIV-1 integration sites were enriched near all non-B DNA features except A-phased, triplex and Z-DNA. SIV and FIV sites were enriched near inverted repeats (100–150 bp away), mirror repeats and short tandem repeats (350–400 bp away). HTLV-1 sites were enriched near A-phased (<50 bp away), cruciform (150–200 bp away) and inverted repeats (within the feature). FV sites were enriched near G4 and Z-DNA (<50 bp away) and triplex DNA (300–350 bp away). MLV sites were enriched near G4 (300–500 bp away), triplex (within and 50–200 bp away) and Z-DNA (150–200 bp away). ASLV sites were enriched in inverted repeats (200–250 bp away), slipped (150–200 bp away) and triplex DNA (150–200 bp and 450–500 bp away). MMTV sites were enriched in short-tandem repeats and slipped DNA (300–350 bp away). Pairwise analyses of the different integration site profiles (based on fold enrichment and depletion values within 500 bp of each feature) showed that HIV-1 and FV had the most similar preferences for targeting non-B DNA, whereas SIV and FIV had the least similarity to the other retroviruses (Figure 2C). Taken together, these data show that evolutionarily diverse retroviruses target non-B DNA for integration and that each retrovirus exhibits distinct preferences for certain non-B DNA features.

### 3.4. HIV-1 Integration Site Profiles Differ between In Vitro- and In Vivo-Derived Datasets

**Figure 3:**
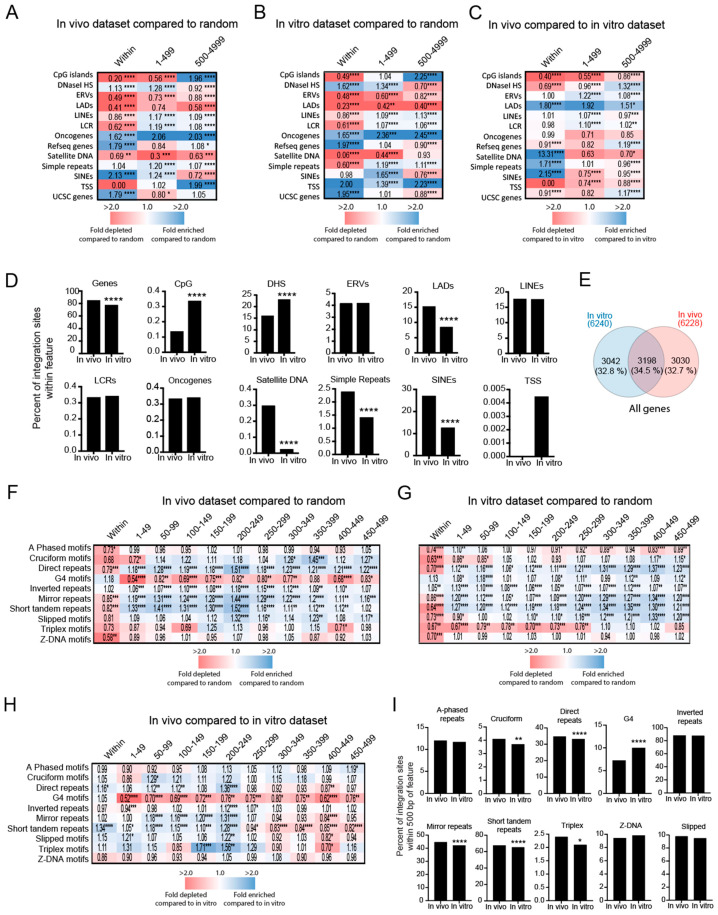
Integration site profiles differ between in vitro- and in vivo-derived datasets. (**A**–**C**) Heatmaps illustrating the fold-enrichment or depletion of unique integration sites (compared to the matched random control) near common genomic features from in vivo-derived datasets (n = 22,372 sites) (**A**), in vitro-derived datasets (n = 67,659 sites) (**B**), or a comparison of in vivo-derived with in vitro-derived sites (**C**). Numbers represent the fold-change in the percentage of integration sites. (**D**) Comparison of the percentage of integration sites within 5000 bp of common genomic features between in vitro- and in vivo-derived datasets. (**E**) Venn diagram showing the number of genes targeted for integration that were unique, or shared by, the in vivo- and in vitro-derived integration site datasets. (**F**–**H**) Heatmaps illustrating the fold-enrichment of unique integration sites compared to the matched random control near non-B DNA from in vivo-derived (**F**) and in vitro-derived (**G**) or a comparison of in vivo-derived with in vitro-derived sites datasets (**H**). (**I**) Comparisons of the percentage of integration sites within 500 bp of non-B DNA between in vitro- and in vivo-derived datasets. Significant differences are denoted by asterisks * *p* < 0.05; ** *p* < 0.01; *** *p* < 0.001; **** *p* < 0.0001) (Fisher’s exact test, two-sided).

Despite a large number of integration site studies from HIV-1 infected individuals recently, many previous integration site studies were performed on infections carried out in vitro [12,61]. To determine if the HIV-1 integration site profiles from in vitro-derived infections differed from those from in vivo-derived infections, we analyzed and compared nine previously published HIV-1 integration site datasets from publicly available databases, totaling 67,659 unique in vitro-derived sites and 22,372 unique in vivo-derived sites (Table S1). As expected, integration sites were significantly enriched in genes, compared to the random controls in both the in vitro- and in vivo-derived datasets; however, only 77% of the total in vivo-derived integration sites were in genes, compared to 84% in the in vitro dataset (*p* < 0.0001; Fisher’s exact test, two-sided) (Figure 3A–D and Table S4). Of the 6240 genes targeted in the in vitro-derived datasets and the 6228 genes targeted in the in vivo-derived datasets, only 34.5% of the genes were targeted by both datasets, indicating some distinct and shared gene preferences for integration between the two datasets (Figure 3E). In addition, integration sites from the in vitro-derived datasets were significantly more enriched in CpG islands and DHS, compared to the in vivo-derived sites (*p* < 0.0001; Fisher’s exact test, two-sided), whereas the in vivo-derived datasets, sites were significantly more enriched in LADs, satellite DNA, simple repeats and SINEs (*p* < 0.0001; Fisher’s exact test, two-sided) (Figure 3A–D and Table S4).

Compared to the in vitro dataset, integration sites in the in vivo-derived dataset were modestly enriched within 250 bp of most non-B DNA except G4 and Z-DNA (Figure 3F–I and Table S4). Notably, in vitro-derived sites were significantly more enriched near G4 DNA than in vivo-derived sites (Figure 3F–I and Table S4). Together, these data show that there are substantial differences in the HIV-1 integration site targeting of genes, CpG islands, DHS, LADs, satellite DNA, simple repeats and SINEs between in vitro-derived and in vivo-derived HIV-1 datasets. Modest but significant differences in preferences were also observed for several non-B DNA features, with G4 DNA being highly favored in the in vitro-derived datasets.

### 3.5. Integration Site Profiles Differ in Individuals Infected with HIV-1 Subtype A, B, C or D

Thus far, integration site profiles have been extensively analyzed for HIV-1 subtype B infections, which represents only ~12% of the infections worldwide. Subtypes A, C and D represent approximately 10%, 47% and 3%, respectively. We asked if the integration site profiles from individuals infected with HIV-1 non-subtype B virus were similar to those infected with subtype B virus. In the integrase enzyme, the amino acid difference between subtypes is as high as 16% (subtype B vs. C) but typically less than 8% in integrase of HIV-1 isolates of a specific subtype. This level of amino acid diversity is the highest among the enzymes encoded by the HIV-1 pol gene. The greatest diversity within HIV-1 integrase is found in the C-terminal domain (CTD), which is involved in genomic DNA binding. Thus, it is reasonable to propose that we would observe differences in the integration site profiles between different HIV-1 subtypes. To address this, genomic DNA was isolated from peripheral blood mononuclear cells (PBMCs) from a cohort of women in Uganda and Zimbabwe infected with HIV-1 subtype A, C or D and used to generate integration site libraries. Integration site profiles were generated from a total of 48 infected individuals (16 subtype A, 19 subtype C and 13 subtype D) and compared to the integration site profile from at least 25 individuals infected with subtype B virus generated from previously published (‘HIV-1 in vivo-derived’) datasets (Tables S1, S5 and S6).

**Figure 4:**
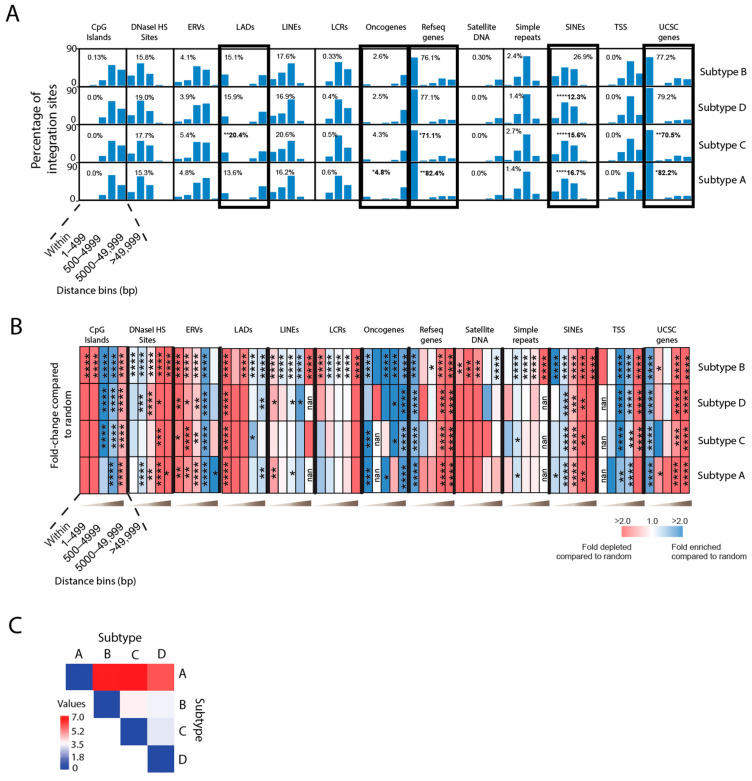
HIV-1 subtype A, B, C and D have different integration site preferences for genomic features. (**A**) Comparison of the percentage of integration sites in vivo near common genomic features between HIV-1 subtypes A, B, C and D. Inset numbers represent the percentages of total integrations directly within the feature. Statistical comparisons were performed with respect to subtype B. Significant differences are denoted by asterisks * *p* < 0.05; ** *p* < 0.01; *** *p* < 0.001; **** *p* < 0.0001 (Fisher’s exact test, two-sided). (**B**) Heatmaps depicting the fold enrichment or depletion of integration sites near common genomic features compared to the matched random control. Darker shades represent higher fold-changes in the ratio of integration sites to matched random control sites. Distance bins in A and B represent the distance of the integration sites in base pairs away from the genomic feature. (**C**) Pairwise analysis was performed on the retroviral integration site profile preferences (based on fold enrichment and depletion values within 5000 bp of each feature) using the Euclidean distance as the measurement method (Heatmapper) [56]. Weaker relationships between retroviral integration site profiles are indicated by darker red color in the pairwise distance matrix, whereas stronger relationships are indicated by darker blue color. Significant differences are denoted by asterisks * *p* < 0.05; ** *p* < 0.01; *** *p* < 0.001; **** *p* < 0.0001 (Fisher’s exact test, two-sided).

Overall, the four different subtype infections yielded similar distributions of integration sites near common genomic features with some notable differences (Figure 4A,B and Table S7). As previously observed with HIV-1 subtype B infections, integration sites from all HIV-1 subtype viruses were enriched in genes (subtype A: 82%, B: 77%, C: 71%, D: 78%) (Figure 4A,B and Table S7). Notably, subtype A had significantly more integration sites in genes compared to subtype B, and subtype C had significantly less integrations in genes compared to subtype B (*p* < 0.05; Fisher’s exact test, two-sided) (Figure 4A and Table S7). Similar to subtype B, subtypes A, C and D disfavored integration into LADs (Figure 4B and Table S7); however, subtype C had significantly more integrations into LADs than subtype B (20.4% versus 15.1% respectively) (Figure 4A and Table S7). All four subtypes also exhibited enriched integration into oncogenes (Figure 4B and Table S7), with subtype A exhibiting significantly more than subtype B (4.8% versus 2.6%) (Figure 4A and Table S7). Moreover, subtypes A, C and D exhibited significantly less integration into SINEs compared to subtype B (16.7%, 15.6% and 12.3% versus 26.9%, respectively) (*p* < 0.0001; Fisher’s exact test, two-sided) (Figure 4A and Table S7). Pairwise analyses of the different integration site profiles showed that subtypes C and D, and B and D, shared the most similarity to each other, and subtype A differed the most from the other subtypes (Figure 4C).

**Figure 5:**
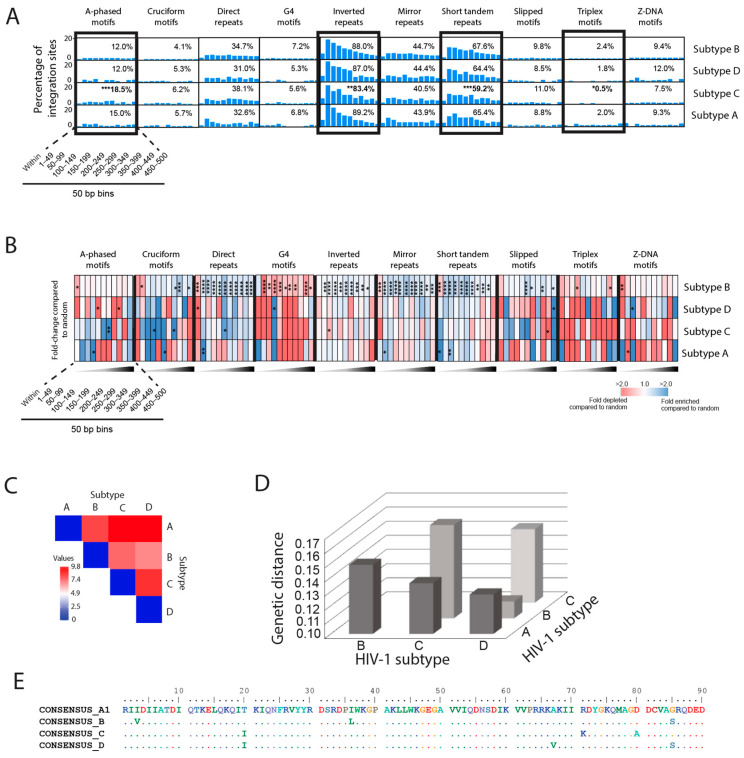
HIV-1 subtype A, B, C and D have different integration site preferences for non-B DNA. (**A**) Comparison of the percentage of integration sites in vivo near non-B DNA features between HIV-1 subtypes A, B, C and D. Inset percentages refer to the total integrations within 500 bp of the feature. Statistical comparisons were performed with respect to subtype B. Significant differences are denoted by asterisks * *p* < 0.05; ** *p* < 0.01; *** *p* < 0.001; **** *p* < 0.0001 (Fisher’s exact test, two-sided). (**B**) Heatmaps depicting the fold enrichment or depletion of integration sites near non-B DNA compared to the matched random control. Darker shades represent higher fold-changes in the ratio of integration sites to matched random control sites. Bins in A and B represent the distance of the integration sites in base pairs away from the non-B DNA feature. (**C**) Pairwise analysis was performed on the retroviral integration site profile preferences (based on fold enrichment and depletion values within 500 bp of each feature) using the Euclidean distance as the measurement method (Heatmapper) [56]. Weaker relationships between retroviral integration site profiles are indicated by darker red color in the pairwise distance matrix, whereas stronger relationships are indicated by darker blue color. Significant differences are denoted by asterisks * *p* < 0.05; ** *p* < 0.01; *** *p* < 0.001; **** *p* < 0.0001 (Fisher’s exact test, two-sided). (**D**) Estimates of evolutionary divergence over sequence pairs between groups. The number of amino acid substitutions per site from averaging over all sequence pairs between groups are shown. Analyses were conducted using the Poisson correction model. This analysis involved 486 amino acid sequences. The coding data was translated assuming a standard genetic code table. All ambiguous positions were removed for each sequence pair (pairwise deletion option). There was a total of 265 positions in the final dataset. Evolutionary analyses were conducted in MEGA X. (**E**) Amino acid alignment of the C-terminal domain of HIV-1 integrase from subtypes A, B, C and D.

Our analysis of the non-B DNA integration site profiles from the different subtype viruses showed enriched integration near most non-B DNA (Figure 5A,B and Table S8). Comparison of the percentage of sites falling within 500 bp of each non-B DNA feature showed that subtype C had significantly more sites near A-phased DNA compared to subtype B (18.5% versus 12.0%, respectively) and significantly fewer sites near inverted repeats, short tandem repeats and triplex motifs compared to subtype B (83.4%, 59.2% and 0.5% versus 88.0%, 67.6% and 2.4%, respectively) (Figure 5A and Table S8). Our analysis of the distribution of sites (in 50 bp bins) within the 500 bp window around each non-B DNA feature revealed some notable observations. Subtypes A and C had significant enrichment of integration sites within 350 bp of A-phased and cruciform DNA (Figure 5B and Table S8). Subtype A exhibited significant enrichment of sites within 100 bp of direct repeats, mirror repeats and short tandem repeats. Subtype C exhibited significant enrichment of sites 200–250 bp away from direct repeats, whereas subtype D exhibited significant enrichment of sites 100–150 bp away from G4 motifs, 450–500 bp away from slipped motifs and 50–100 bp away from Z-DNA motifs (Figure 5B and Table S8). Pairwise analysis of the different non-B DNA integration site profiles showed that the profiles of all subtypes differed substantially from each other, with subtypes B and D showing more similarity to each other compared to the other subtypes (Figure 5C). Intriguingly, the similarity in integration preference for common genomic features (as observed in Figure 4C) and non-B DNA between subtypes B and D correlated in part to the sequence distance between integrase coding regions of subtypes A, B, C, and D, with B and D being most genetically similar (Figure 5D,E). However, it is important to stress that any differences in integration profiles could also relate to discreet single amino acid differences between subtypes that could affect DNA binding and/or protein-protein interactions of the PIC.

Together, these data show that the integration site profiles from individuals infected with HIV-1 subtype B differ from those infected with non-subtype B virus. Subtypes B and D exhibited the most similar profiles overall. Subtype A tended to exhibit a more transcriptionally active profile compared to subtype B (e.g., favored genes and oncogenes), and subtype C exhibited a more transcriptionally silent profile than subtype B (e.g., favored LADs and disfavored genes). All subtypes favored integration near non-B DNA but differed in their preference for certain types of non-B DNA.

### 3.6. Integration Site Hotspots Are Shared between HIV-1 Subtypes

**Figure 6:**
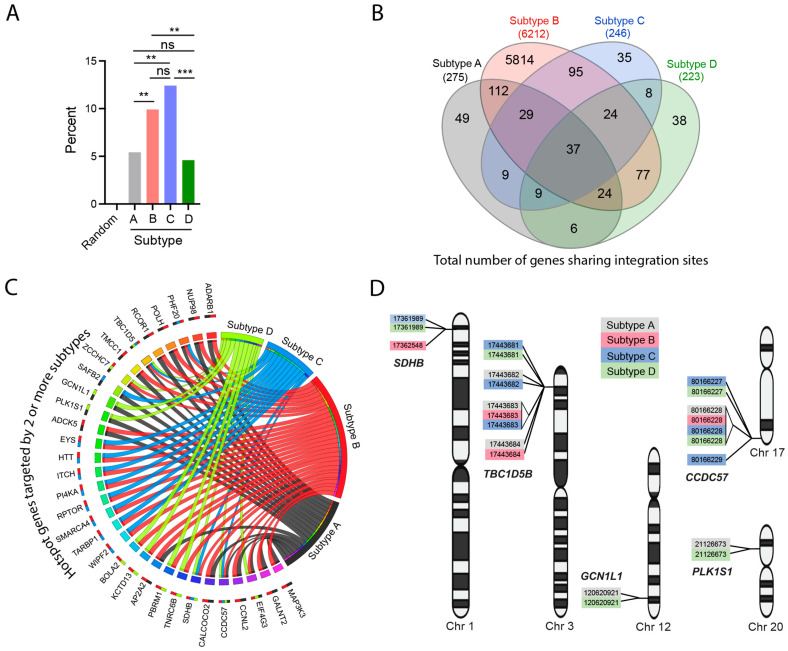
Integration hotspots identified from individuals infected with different HIV-1 subtypes. (**A**) 1000 bp windows of genomic DNA hosting two or more integration sites (“hotspots”) were quantified for each HIV-1 subtype and summarized as the percentage of the total number of integration sites falling within a hotspot. **, *p* < 0.01; ***, *p* < 0.001; ns, not significant; Fisher’s exact test, two-sided. (**B**) All genes targeted by each HIV-1 subtype were filtered and compared to each other to identify genes uniquely targeted by each subtype or genes targeted by more than one subtype. The Venn diagram shows the number of unique and shared genes between the different subtypes. (**C**) All genes hosting two or more integration sites (‘gene hotspots’) were filtered for each HIV-1 subtype and compared to each other to identify genes targeted by two or more subtypes. The ribbons emerging from each subtype in the Circos plot connect to the genes (each represented by a different colored box) shared by other subtypes. The multi-colored bars next to each gene name summarize the subtypes targeting those genes. (**D**) All genes hosting two or more integration sites that were <1000 bp apart (‘gene super-hotspots) were filtered for each HIV-1 subtype and compared. The chromosomal location and gene names of the gene hotspots targeted by two or more subtypes are shown at their approximate chromosomal location on their respective human chromosome. Identical chromosomal locations indicate shared integration sites between the different datasets.

The concept of an HIV-1 integration ‘hotspot’ was introduced to describe areas of the genome where integrations accumulate more than expected by chance in the absence of any selection process [9,62]. We analyzed 1000 bp windows containing two or more integration sites from each HIV-1 subtype dataset. This yielded a total of 2102 hotspots in 21,506 total integration sites. The percentage of hotspots were similar between subtypes C (12.4%; 46/372 sites) and B (9.9%; 2024/20,499 sites) (*p* > 0.05; Fisher’s exact test, two-sided), and between subtypes A (5.4%; 19/352 sites) and D (4.6%; 13/283 sites) (*p* > 0.05; Fisher’s exact test, two-sided) (Figure 6A). For comparison, the MRC yielded only two hotspots out of 2982 sites (0.07%). Our analysis of all of the genes targeted by the four subtypes showed that there were many genes that were uniquely targeted by each subtype (Figure 6B and Table S9). In addition, several genes were targeted by multiple subtypes, including 37 genes that were targeted by all four subtypes. To determine if some of these genes were targeted more often than others, we analyzed all genes hosting two or more integration sites (‘gene hotspots’) for each HIV-1 subtype. Thirty-two gene hotspots were targeted by two or more subtypes (Figure 6C). Notably, *TBC1D5* and *CCDC57* were targeted by all four subtypes and *PHF20*, *ZCCHC7*, *SDHB* and *EIF4G3* were targeted by three different subtypes.

We then analyzed these gene hotspots hosting two or more integration sites that were <1000 bp apart (‘gene super-hotspots’) and identified five genes (*SDHB*, *TBC1D5B*, *GCN1L1*, *CCDC57* and *PLK1S1*) that were targeted by multiple subtypes (Figure 6D). Inspection of the chromosomal locations of the integration events revealed multiple identical integration sites shared by two or more subtypes (Figure 6D). Notably, chr3:17443683 was shared by subtypes A, B and C, and chr17:80166228 was shared by all four subtypes. Together, these data show that certain genomic locations are highly and precisely targeted for integration by different HIV-1 subtypes.

### 3.7. Integration Site Hotspots Are Located in Slipped DNA Motifs

**Figure 7:**
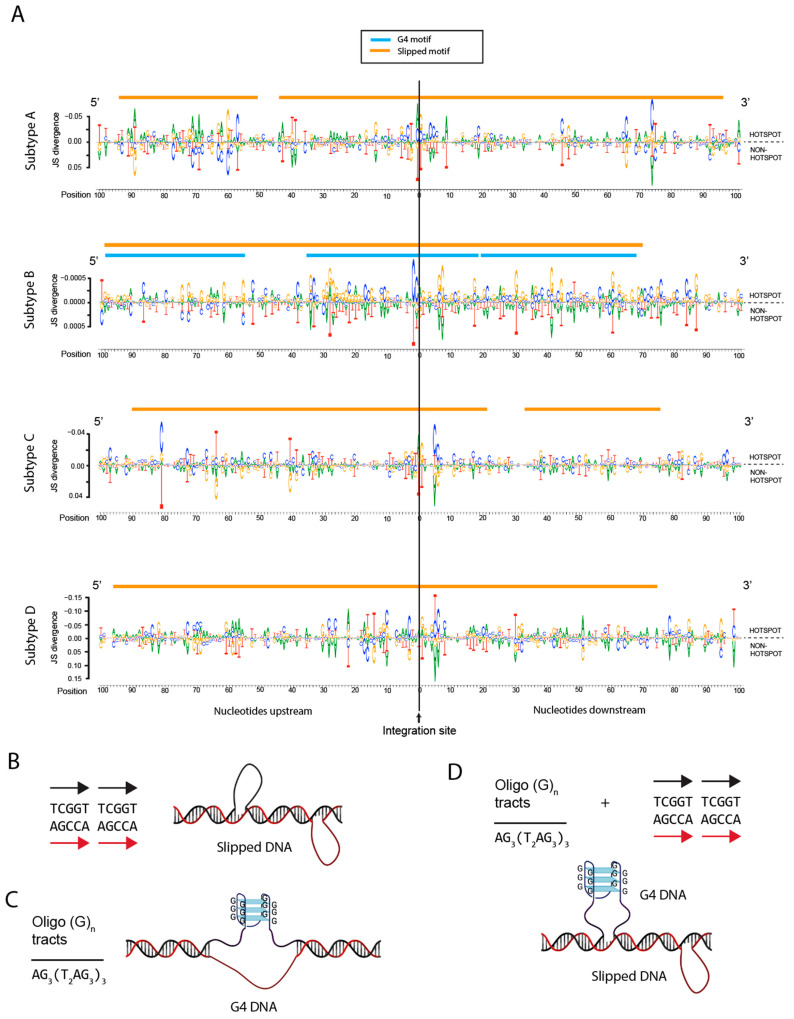
Integration site hotspots for HIV-1 subtypes A, B, C and D are located in non-B DNA. (**A**) Genomic sequences were extracted from a window of 100 nucleotides upstream and 100 nucleotides downstream of each integration site. Sequences from integration sites located in hotspots were compared to sequences from sites not located in hotspots using DiffLogo. Consensus sequences were analyzed for the presence of non-B DNA motifs and represented by colored lines above each DiffLogo image (orange, slipped DNA motif; blue, G4 DNA motif). The top half of each DiffLogo represents sequences from hotspots and the lower half represents sequences from non-hotspots. (**B**–**D**) Example sequences and graphical representations of slipped DNA (**B**), G4 DNA (**C**) and slipped plus G4 DNA (**D**) features.

To identify potential local genomic sequences that may serve as ‘beacons’ for increased integration, we compared two pools of integrations sites: the first pool contained integration sites located in all hotspots (two integration sites that were <1000 bp apart regardless of being located in a gene or not) and the second pool contained integration sites not located in hotspots. We then extracted nucleotide windows of 100 bp upstream and 100 bp downstream of each integration site and used DiffLogo to identify motifs that predominate in the pool of hotspot sites. DiffLogo provides a visualization of pair-wise differences between DNA motifs [63]. It presents the characteristics of each motif position by the stack height and symbol height within a stack. The stack height is proportional to the degree of distribution dissimilarity (Jensen–Shannon divergence), whereas the symbol height is proportional to the degree of differential symbol abundance. Overall, subtype D exhibited the largest divergence in sequence preferences within the 200 bp window, followed by subtypes A and C, with subtype B yielding the least sequence divergence (Figure 7A). Our analysis of the 200 bp consensus hotspot sequence motifs (nucleotides with the largest degree of differential symbol height within each stack in the upper portion of each graph in Figure 7A) revealed the presence of slipped DNA motifs spanning almost the entire 200 bp window for each subtype (Figure 7A,B). In addition, subtype B also had three G4 DNA motifs that overlapped the slipped DNA motifs (Figure 7A–D). Together, these data show that integration sites located in hotspot regions in all of the subtypes are located in slipped DNA motifs, with subtype B sites also falling within and/or near a G4 DNA motif.

## 4. Discussion

Here we showed that although evolutionarily divergent retroviruses exhibit distinct integration site profiles, all retroviruses target non-B DNA for integration. We also showed that there are important differences in the integration site profiles between in vivo- and in vitro-derived HIV-1 datasets, but that the initial targeting of non-B DNA (except for G4 DNA) during acute infection does not appear to differ substantially. Comparison of the integration site profiles of different HIV-1 subtypes revealed that there are significant differences when comparing subtype B and non-subtype B infections. Notably, subtype C, which comprises ~47% of HIV-1 infections worldwide, exhibited a more transcriptionally silent profile (e.g., increased integration in LADs and reduced integration in genes) and exhibited the largest differences in preferences for non-B DNA. Despite these differences, we identified several integration site hotspots that are shared between the different subtypes, all of which were located in a slipped DNA motif.

As previously observed with HIV-1, the distribution of integration sites within 500 bp of non-B DNA features for all retroviruses was often clustered in discrete distances away from the feature [46]. One explanation could be that the PIC is first attracted to the non-B DNA structure where, due to the size of the PIC, it then integrates next to the feature. Other explanations for the heterogeneity could be differences in the cellular transcriptional profiles that could affect the formation of non-B DNA structures, the adjacent nucleosome occupancy, and/or non-B DNA-binding proteins that generate steric constraints for integration at those locations. Moreover, genetic polymorphisms (e.g., insertions/deletions) near non-B DNA motifs may also contribute to differences in the distance of the integration sites to the features in infected individuals. For example, repeat expansion may alter the location and size of slipped or G4 DNA loops (Figure 7B–D).

All retroviruses exhibited enriched integration near non-B DNA with slipped DNA serving as integration site hotspots for HIV-1 subtypes A, B, C and D. Slipped DNA motifs at integration site hotspots are not limited to HIV-1. Other studies identified consensus motifs for a variety of other retroviruses with no previously identified commonalities among the different motifs [11,42,64]. An example of some of these motifs include: HTLV-1: TTTTTAAGTCCTTTTCCACTTTAATT; HIV-1: TTTTTTTTT(N)GTTACCTAATTTTTT; MLV: TTTTTATTTCCTATCA(N)CTTTTAT; ASLV: TTTTTTCTATCTTTTCTAACTTTTTT; PFV: TTT(N)(N)T(N)CTTGCCACCACCCTTT(N)CT; and FIV: ATATTAAATTTTTAAAAATAGTTATTATTTTAAATATTTA. Our re-analysis of these previously identified consensus motifs revealed that these motifs are all indeed slipped DNA motifs, supporting our findings that non-B DNA are key features targeted by all retroviruses and that slipped DNA represent integration site hotspots. Slipped DNA structures are remarkably stable structures that form at sequences containing consecutive repeat sequences where one repeat unit misaligns with the second repeat unit on the opposite DNA strand (Figure 7B) [65].

How retroviral PICs recognize slipped DNA and other non-B DNA for integration is not yet fully understood. Many non-B DNA motifs are enriched near sites of genomic variability and are targets for preferential homologous recombination (>20-fold) in human cells [66,67,68]. It is possible that integrase itself, components of the PIC and/or host proteins can bind non-B DNA directly and promote integration into these regions. We recently showed that PIC-binding host factors APOBEC3, LEDGF/p75 and CPSF6 influence the distribution of integration sites near non-B DNA features, potentially indicating that a host-directed mechanism is involved [39,46]. Intriguingly, APOBEC3G binding and deamination hotspots also comprise slipped DNA motifs, potentially helping direct the PIC to slipped DNA [69,70]. Moreover, HIV-1 integrase has been shown to bind directly to G4 DNA; therefore, integrase itself may also contribute to the binding of non-B DNA for integration [71]. Interestingly, we detected a G4 motif at HIV-1 integration site hotspots that overlaps a slipped DNA motif. It is currently unknown which non-B DNA feature would predominate at that position, but it is possible that both could be present depending on the length of the consecutive repeat sequences driving formation of the slipped strand structure on the opposite DNA strand (Figure 7D). Recently, we showed that G4 DNA also influences productive and latent HIV-1 integration and the reactivation potential of HIV-1 [46]. Our finding that G4 DNA motifs are highly targeted during acute infection (in vitro-derived datasets) supports the importance of G4 DNA as a targeted feature for integration. A more detailed biochemical and structural characterization of PICs with non-B DNA is needed to better understand the involvement of non-B DNA at the site of integration and whether the duo of G4 and slipped DNA features at integration site hotspots is a unique property of HIV-1.

Analysis of integration site profiles after acute infection showed that HIV-1, FIV and SIV had the strongest preference for integrating into transcriptionally active regions of the genome (e.g., genes), whereas MMTV and FV had the strongest preference for transcriptionally inactive regions (e.g., LADs, satellite DNA and SINEs). The targeting of transcriptionally active regions of the genome maximizes provirus expression and helps establish infection and spread early in infection [9]. However, this comes with a cost to the virus because the expression of viral proteins during replication can trigger the host-dependent killing of the infected cells via the immune system or killing via virus-induced cytopathic effects. As one potential strategy for long-term survival and escape from the immune system, retroviruses have also acquired the ability to insert their genomes into more transcriptionally repressive regions of their host’s genome [72,73,74,75,76]. These regions of the genome are characterized by several features. For example, LADs represent a repressive chromatin environment tightly associated with the nuclear periphery [59,60]. SINEs (e.g., Alu repeats) and other transposed sequences are known to serve as direct silencers of gene expression due to their repressed chromatin marks (histone H3 methylated at Lys 9) [77,78]. In addition, non-B DNA structures, such as G4, cruciform, triplex and Z-DNA have been shown to silence the expression of adjacent genes [68,79,80,81,82,83,84,85,86,87,88]. Ancient retroviruses, some of which have been co-evolving with their hosts for hundreds of millions of years (e.g., ERVs and FV), are commonly integrated in transcriptionally silent regions of the genome away from genes [89,90,91], and as shown herein for FV, LADs, satellite DNA, SINEs, G4, triplex and Z-DNA. As such, the host benefits with a lower risk of disease. This is seen with FV which persists in their primate hosts in the absence of disease, and MMTV which typically does not cause cancer unless it integrates near an oncogene [92].

By integrating into transcriptionally repressive regions of the genome, retroviruses can minimize their expression and avoid detection by the immune system after the high virus-expressing cells are destroyed. In strong support of this strategy, recent work has shown that most intact HIV-1 proviruses in infected individuals who naturally control infection (i.e., elite controllers), and individuals on long-term suppressive antiretroviral therapy, are located in transcriptionally repressive features [60,72,73,74,75,76,93]. Our comparison of the in vitro (acute HIV-1 infection) versus in vivo (long-term HIV-1 infection) integration site profiles supports the findings by Einkauf et al. (2019), Jiang et al. (2020), Einkauf et al. (2022) and Lian et al. (2023) showing that in vivo-derived sites are significantly more enriched in transcriptionally repressive features of the genome (e.g., LADs, SINEs, satellite DNA, G4, cruciform and triplex DNA) compared to the in vitro-derived sites (Figure 3). A limitation of our study is that we did not differentiate between intact and non-intact retroviral genomes. Since RNA and/or protein expression from non-intact HIV-1 proviruses also plays an important role in immune escape, persistence and pathogenesis (reviewed in [94]), it will be interesting to determine if there are integration site biases for non-B DNA features between intact and non-intact proviruses, and whether non-B DNA features are responsible for attracting PICs to transcriptionally repressive regions.

It is not fully understood if integration into transcriptionally silent regions of the genome is driven by retroviruses, the host or both; however, current evidence supports the latter of the three. For example, the host antiviral proteins APOBEC3 appear to promote a transcriptionally silent integration site profile for HIV-1 that is away from genes and enriched in SINEs [39]. This suggests that the host contributes to this silent phenotype, perhaps as a protective antiviral mechanism. However, there is evidence that the virus also contributes to this silent phenotype. For example, a point mutation in HIV-1 integrase was shown to redirect the proviral integration into centromeric repeats [95], an event known to be enriched in latently infected cells and in elite controllers [76]. Moreover, we showed here, for example, that individuals infected with HIV-1 subtype C have a strong transcriptionally silent integration site profile compared to subtype A, which exhibits a strong transcriptionally active profile.

Much of our knowledge of HIV-1 integration site selection has come from studies using subtype B virus. Our comparative analyses showed several differences in the integration profiles of different subtype viruses. Notably, integration sites from subtype A infection showed a much stronger enrichment in transcriptionally active regions of the genome, characterized by increased integration sites located in genes and oncogenes and decreased sites in genomic features associated with transcriptional silencing, such as LADs and SINEs. Conversely, integration sites from a subtype C infection showed a much stronger enrichment in transcriptionally inactive regions of the genome, characterized by decreased integration sites in genes and increased sites in LADs and SINEs. These unique profiles also correlated with unique preferences for integration near non-B DNA features. The mechanism underlying these striking differences in the integration site profiles is currently unknown. One mechanism may involve amino acid differences in HIV-1 integrase between the different subtypes that could affect the protein composition and/or genomic targeting of HIV-1 PICs. In the integrase coding region, amino acid differences between subtypes are as a high as 16% (subtype B vs. C) but typically less than 8% integrase diversity among HIV-1 isolates of one subtype [13,96,97]. This level of amino acid diversity is the highest among the enzymes encoded by the HIV-1 *pol* gene. The greatest diversity within HIV-1 integrase is found in the CTD, which is involved in genomic DNA binding. Thus, it is reasonable to suspect that HIV-1 subtypes may target different non-B DNA motifs with different selectivity. Despite the multiple substitutions that segregate HIV-1 variants of each subtype, there may be specific amino acid differences in one subtype versus another that impact preference for integration sites. For example, the isoleucine found at position 20 in subtype C and D versus the threonine in subtype A and B in the integrase CTD may relate the greater similarity of integration site selection of subtypes B and D (Figure 5D,E). In addition, amino acid differences in subtypes A and C, such as lysine at position 71 and/or alanine at position 80 of subtype C integrase CTD, may confer alternative DNA targeting properties of the PICs towards transcriptionally active versus silent regions of the genome. Despite the closer genetic relationship between HIV-1 subtype B and D across the genome, similar genetic distances separating subtypes A, B, C and D are found within the integrase coding region. Polymorphisms in HIV-1 integrase have been reported that retarget integration away from gene dense regions, which also correlated with increased disease progression and virulence [95,98]. This suggests that characteristics of integrase itself is a driver of integration site selection in retroviruses. We are currently exploring if subtype-specific polymorphisms in integrase may account for differences in the targeting of transcriptionally silent regions of the genome and of non-B DNA.

Recently, we showed that human APOBEC3 increased HIV-1 insertions into SINEs in a dose-dependent manner [39]. Interestingly, we showed here that integration sites from non-subtype B infections were all significantly reduced in SINEs compared to subtype B infections. This begs the question, does differential HIV-1 Vif activity among the different subtypes impact APOBEC3 levels and subsequently APOBEC3-mediated integration site targeting of SINEs and other DNA features? Indeed, Binka et al. (2012) showed that the activity spectra of Vif, especially towards the A3H haplotype II, showed differences in their abilities to inhibit APOBEC3 [99]. It will be interesting to learn if Vif levels and/or Vif polymorphisms impact integration site targeting, and whether the ability of other retroviruses to alter APOBEC3 levels impacts their integration site targeting. Given that all of the non-subtype B infections in our study were derived from individuals in Uganda or Zimbabwe, it is also possible that genetic polymorphisms in APOBEC3 may impact APOBEC3 function and/or Vif-induced degradation of APOBEC3 proteins, leading to differential APOBEC3-mediated integration site targeting.

## 5. Conclusions

We identified non-B DNA as a feature that is targeted differentially by evolutionarily diverse retroviruses. We have also presented the first comparative look at the integration site profiles of HIV-1 subtype A, C and D viruses and showed that they differed from HIV-1 subtype B profiles overall but shared similar integration site hotspots located in slipped DNA. Together, these data highlight important similarities and differences in retroviral integration site targeting that can be used in future studies to better understand the evolution of retroviral integration site targeting and how these viruses integrate into our genomes for long-term survival. It will be important to investigate the impact of non-B DNA features on the expression of nearby proviruses and their contributions to productive and latent integrations. The identification and characterization of proteins that influence targeting of non-B DNA by retroviral preintegration complexes will also further our understanding of integration site selection and possibly open new avenues for drug targets and the design of safer and more efficient retroviral gene therapy vectors.

## Data Availability

Integration site locations in the human genome were obtained from the GRCh37/hg19 database (https://hgdownload.soe.ucsc.edu/downloads.html). The integration site bedfiles are provided in Table S6.

## Supplementary Materials

The following supporting information can be downloaded at: https://www.mdpi.com/article/10.3390/v15020465/s1. Table S1: List of retrovirus integration site datasets used in this study. Table S2: Integration site profile of evolutionarily diverse retroviruses in common genomic features. Table S3: Integration site profile of evolutionarily diverse retroviruses in non-B DNA features. Table S4: Comparison of the integration site profiles of in vitro- and in vivo-derived datasets. Table S5: Clinical profile of non-subtype B study participants used in this study. Table S6: Integration sites of HIV-1 subtypes A, C and D. Table S7: Integration site profile of subtypes A, B, C and D in common genomic features. Table S8: Integration site profile of subtypes A, B, C and D in non-B DNA features. Table S9: List of genes targeted by two or more HIV-1 subtypes.
